# The lived experience of resilience in families of children with cancer: a meta-aggregative synthesis of qualitative studies

**DOI:** 10.3389/fpsyt.2026.1856540

**Published:** 2026-08-21

**Authors:** Chunyan Li, Chuanying Zhang, Sijing Peng, Yixin Wang, Qianqian Hu

**Affiliations:** 1 School of Nursing, Anhui University of Chinese Medicine, Hefei, Anhui, China; 2 Laboratory of Geriatric Nursing and Health, Anhui University of Chinese Medicine, Hefei, Anhui, China; 3 School of Social and Behavioral Sciences, Nanjing University, Nanjing, Jiangsu, China

**Keywords:** children with cancer, family resilience, caregivers, meta-aggregative synthesis, qualitative studies

## Abstract

**Background:**

Childhood cancer imposes substantial psychological, social, and practical challenges on families, often disrupting emotional stability, caregiving roles, financial security, and family functioning. Family resilience has increasingly been recognized as a dynamic process that supports adaptation under prolonged adversity. However, qualitative evidence regarding how families develop and sustain resilience in the context of childhood cancer remains fragmented.

**Objective:**

This qualitative systematic review aimed to synthesize existing qualitative evidence on family resilience among families of children with cancer and to identify key resilience processes, coping strategies, and influencing factors associated with adaptive family functioning.

**Methods:**

This review was conducted in accordance with the Preferred Reporting Items for Systematic Reviews and Meta-Analyses (PRISMA) guidelines and the Joanna Briggs Institute (JBI) methodology for qualitative evidence synthesis. Relevant studies were identified through systematic database searches using structured combinations of terms related to childhood cancer, family resilience, caregiving, and parental adaptation. Eligible qualitative studies were critically appraised using the JBI Critical Appraisal Checklist for Qualitative Research. Findings were synthesized using meta-aggregation to generate categories and broader synthesized findings.

**Results:**

Four synthesized findings emerged. First, family resilience involved emotional and cognitive transformation, in which families initially experienced fear, uncertainty, guilt, helplessness, and psychological disruption, followed by emotional adjustment, acceptance, and reconstruction of hope and meaning. Second, behavioral adaptation and family reorganization represented critical resilience processes, including redistribution of caregiving responsibilities, restructuring of daily routines, and strengthened family cohesion. Third, external support functioned as an important protective factor in resilience development, with healthcare professionals, peer networks, and relatives providing emotional, informational, and practical support, although financial strain, caregiving intensity, and emotional exhaustion could limit access to such resources. Fourth, coping strategies employed by parents influenced resilience sustainability, with problem-focused coping, emotional regulation, and meaning-making facilitating adaptation, whereas avoidance and passive withdrawal were associated with weaker long-term adjustment.

**Conclusions:**

Family resilience in childhood cancer should be understood as a dynamic and multidimensional adaptation process shaped by emotional transformation, behavioral reorganization, external support, and coping strategies. Family-centered interventions that strengthen resilience-promoting coping, improve access to support systems, and facilitate adaptive family functioning may enhance long-term psychosocial outcomes in pediatric oncology care.

**Systematic review registration:**

https://www.crd.york.ac.uk/prospero/display_record.php?ID=CRD42024551729, identifier CRD42024551729

## 1 Introduction

Childhood cancer remains a major global health challenge that affects not only children but also the entire family system. Compared with many acute pediatric illnesses, childhood cancer is often characterized by prolonged treatment, uncertainty regarding prognosis, recurrent hospitalization, financial burden, caregiving complexity, and persistent psychological stress (1, 2). These challenges frequently disrupt family routines, alter parental roles, and influence emotional well-being, interpersonal relationships, and long-term family functioning.

The diagnosis of childhood cancer often produces immediate emotional shock for parents and caregivers. Families may experience fear, helplessness, guilt, uncertainty, anticipatory grief, and concerns regarding treatment outcomes. Beyond emotional burden, caregiving responsibilities frequently intensify during treatment, requiring substantial behavioral adaptation. Parents may need to reorganize employment, redistribute household roles, manage financial pressure, and sustain caregiving continuity while simultaneously supporting the emotional needs of the child and other family members.

In this context, family resilience has emerged as an important concept in pediatric oncology. Family resilience is increasingly understood as a dynamic process through which families adapt, reorganize, and maintain functional stability under prolonged adversity (3, 4). Rather than representing an individual trait, family resilience reflects multidimensional interactions involving emotional regulation, collective coping, relational support, adaptive restructuring, and meaning-making (5–7).

Existing studies have explored parental coping, psychosocial burden, caregiving experiences, and adaptation among families of children with cancer. However, qualitative findings remain dispersed across different cultural contexts and healthcare systems. While individual studies have described emotional distress, caregiving burden, and support needs, broader resilience processes that explain how families adapt and sustain functioning have not been sufficiently synthesized.

A qualitative synthesis is particularly important because resilience development is socially embedded, context-dependent, and shaped by lived experience. Integrating qualitative evidence may improve understanding of family adaptation pathways and inform family-centered interventions in pediatric oncology settings.

Therefore, this qualitative systematic review aimed to synthesize qualitative evidence on family resilience among families of children with cancer and identify key resilience processes, coping strategies, and contextual factors influencing resilience development and sustainability.

In this review, family resilience is conceptualized according to Walsh’s family resilience framework, which emphasizes adaptive belief systems, organizational patterns, and communication processes that enable families to withstand and recover from adversity. Coping strategies, emotional adjustment, and post-traumatic growth are considered resilience-related processes rather than synonymous constructs.

## 2 Methods

### 2.1 Design

This study employed a qualitative systematic review using the Joanna Briggs Institute (JBI) meta-aggregation approach to synthesize evidence on resilience among family caregivers of children with cancer (8, 9). JBI meta-aggregation was selected because it is particularly suitable for synthesizing qualitative findings while preserving the original meaning of primary studies and minimizing reinterpretation, thereby generating practical and clinically applicable recommendations. This methodological orientation was considered appropriate for the present review, as the aim was not to develop new theory, but to systematically consolidate qualitative evidence regarding how resilience is experienced, maintained, and strengthened among families caring for children with cancer across different caregiving contexts.

The JBI meta-aggregation approach is philosophically grounded in pragmatism, which emphasizes the practical utility of synthesized findings for healthcare decision-making and intervention development. In the context of pediatric oncology caregiving, this framework enabled the integration of diverse qualitative evidence while maintaining methodological transparency and applicability to family-centered psychosocial care.

This review was conducted in accordance with the Enhancing Transparency in Reporting the Synthesis of Qualitative Research (ENTREQ) statement (10) and the Preferred Reporting Items for Systematic Reviews and Meta-Analyses (PRISMA 2020) guidelines (11). The review protocol was prospectively registered in the International Prospective Register of Systematic Reviews (PROSPERO; Registration No. (CRD42024551729), which helped ensure methodological transparency and reduce the risk of reporting bias.

To maintain conceptual consistency, all qualitative findings were synthesized using the JBI meta-aggregation process. This approach allowed the review to aggregate findings from heterogeneous caregiving experiences—including treatment, survivorship, and context-specific family adaptation—while preserving the credibility of primary qualitative evidence.

### 2.2 Search strategy

A comprehensive literature search was conducted to identify qualitative studies exploring resilience among family caregivers of children with cancer. To ensure broad coverage across medical, multidisciplinary, psychosocial, and evidence-based health literature, electronic databases including PubMed, Web of Science, ProQuest, Cochrane Library, China National Knowledge Infrastructure (CNKI), and Wanfang Database were systematically searched. These databases were selected because they collectively capture literature relevant to pediatric oncology, family caregiving, psychosocial adaptation, and qualitative healthcare research.

The search covered studies from database inception until December 2025, using predefined search terms (see **Supplementary File S1** for the complete search strategy). A structured search strategy combining controlled vocabulary (where applicable) and free-text terms was used to improve retrieval sensitivity and comprehensiveness. The primary search domains included concepts related to childhood cancer, family caregivers, and resilience. To reduce retrieval bias and improve completeness of evidence identification, the reference lists of all included studies and relevant review articles were manually screened. In addition, grey literature was searched through Google Scholar to identify potentially eligible studies not captured in indexed databases. The study selection process was documented using a PRISMA flow diagram, ensuring methodological transparency and reproducibility.

Because resilience is frequently discussed indirectly in qualitative pediatric oncology literature, studies examining adaptation, coping, family functioning, psychosocial adjustment, meaning-making, and caregiving experiences were also screened for potential relevance. Eligibility was determined based on whether resilience-related processes could be identified conceptually, even when the term “resilience” was not explicitly used by the original authors.

### 2.3 Eligibility criteria

Eligibility criteria were established using the Population–Phenomenon of Interest–Context (PICo) framework to guide study selection for this qualitative systematic review.

Population: The population of interest included family caregivers of children diagnosed with cancer, including parents, guardians, or other primary family members directly involved in long-term caregiving, treatment support, emotional adaptation, or daily management during the child’s cancer trajectory. Studies involving mothers, fathers, or mixed family caregiver groups were eligible for inclusion, as resilience may be experienced differently across caregiving roles and family structures. Children were defined as individuals diagnosed with cancer during childhood or adolescence, based on the age criteria reported in the original primary studies. No restrictions were imposed on cancer subtype, treatment stage, or survivorship phase in order to capture heterogeneous caregiving experiences across diagnosis, treatment, post-treatment adaptation, and long-term survivorship contexts. Neurofibromatosis was included because it is a tumor-predisposition disorder associated with chronic oncological surveillance and cancer-related family caregiving demands.

Phenomenon of Interest: The phenomenon of interest was resilience among family caregivers, including adaptive coping, emotional regulation, meaning-making, family adjustment, psychosocial adaptation, support-seeking, and processes through which caregivers maintained or strengthened functioning under pediatric cancer-related adversity. Studies that explored resilience either explicitly or conceptually through adaptation and coping-related experiences were considered eligible.

Context: Studies conducted across different healthcare, hospital, home-based, survivorship, and community caregiving contexts were included. No geographical restrictions were applied, as resilience may be influenced by healthcare systems, sociocultural expectations, and family support structures.

Study Design: Only qualitative studies were included, such as studies using phenomenology, grounded theory, ethnography, qualitative descriptive design, narrative inquiry, or other recognized qualitative methodologies. Mixed-methods studies were eligible only when qualitative findings could be clearly extracted and analyzed independently.

Studies were excluded if they: focused primarily on children or adolescents with cancer rather than family caregivers; involved caregivers of patients with other chronic or non-oncological diseases; were quantitative-only studies, editorials, commentaries, conference abstracts, protocols, dissertations, or review articles; did not report findings related to resilience, adaptation, or caregiving experiences relevant to the review objective; lacked extractable qualitative data; were not published in English or Chinese.

### 2.4 Study selection

All retrieved records were imported into reference management software for organization and duplicate removal prior to screening. After duplicates were removed, the remaining studies underwent a two-stage screening process in accordance with PRISMA recommendations. In the first stage, titles and abstracts were independently screened by two reviewers to identify potentially relevant studies based on the predefined eligibility criteria. Studies that clearly did not meet the inclusion criteria were excluded at this stage. In the second stage, the full texts of potentially eligible articles were independently assessed by the same reviewers to determine final eligibility. This full-text review was conducted according to the predefined PICo framework, including population, phenomenon of interest, context, and study design criteria. Disagreements between reviewers regarding study eligibility, interpretation of inclusion criteria, or methodological relevance were resolved through discussion and consensus. When necessary, a third reviewer was consulted to minimize selection bias and ensure consistency in decision-making.

### 2.5 Quality appraisal

The methodological quality of all included studies was independently assessed using the Joanna Briggs Institute (JBI) Critical Appraisal Checklist for Qualitative Research. The appraisal focused on several domains, including the congruity between the stated philosophical perspective and research methodology, the alignment between research methodology and data collection methods, the representation and interpretation of participants’ voices, ethical considerations, researcher positioning, and the overall credibility of study findings. Two reviewers independently conducted the quality appraisal for each included study. Any discrepancies in scoring or interpretation were discussed and resolved through consensus. Where disagreement persisted, consultation with a third reviewer was undertaken to ensure consistency and minimize appraisal bias.

### 2.6 Data extraction

Two reviewers independently extracted relevant study information from each eligible article. Extracted data included author(s), year of publication, country of origin, study objective, participant characteristics, caregiver type, child clinical context, study design, data collection method, analytical approach, and key qualitative findings related to resilience among family caregivers of children with cancer. To reduce extraction bias, discrepancies between reviewers regarding extracted content, interpretation of findings, or study-level relevance were resolved through discussion and consensus. Where necessary, consultation with a third reviewer was undertaken.

### 2.7 Data synthesis

Data synthesis was conducted in accordance with the Joanna Briggs Institute (JBI) meta-aggregation methodology. First, all findings reported in the included studies, together with their supporting participant quotations, were extracted and assessed for credibility. Second, findings with similar meanings were grouped into categories based on conceptual similarity. Third, categories were aggregated into higher-order synthesized findings that represented common patterns across studies. Throughout the synthesis process, the review team sought to remain close to the original interpretations of the primary study authors and avoided generating new theoretical explanations beyond the included evidence. This aggregation process enabled the identification of overarching resilience-related experiences while maintaining methodological consistency with the pragmatic philosophy underpinning JBI meta-aggregation.

### 2.8 Confidence in synthesized findings

Confidence in the synthesized qualitative findings was assessed using the ConQual approach (12), as recommended by the Joanna Briggs Institute for qualitative evidence synthesis. The ConQual assessment was based on two core domains: dependability and credibility. Dependability reflected the methodological rigor and consistency of the included studies, which was primarily informed by the results of the JBI Critical Appraisal Checklist for Qualitative Research. Credibility referred to the extent to which the extracted findings were supported by the original study data, including authors’ interpretations and participant quotations. Dependability was assessed based on Questions 2–9 of the JBI Critical Appraisal Checklist. Credibility was determined according to the classification of extracted findings as unequivocal, credible, or unsupported. Downgrading decisions followed JBI ConQual guidance.

## 3 Results

### 3.1 Study search and screening

**Figure 1:**
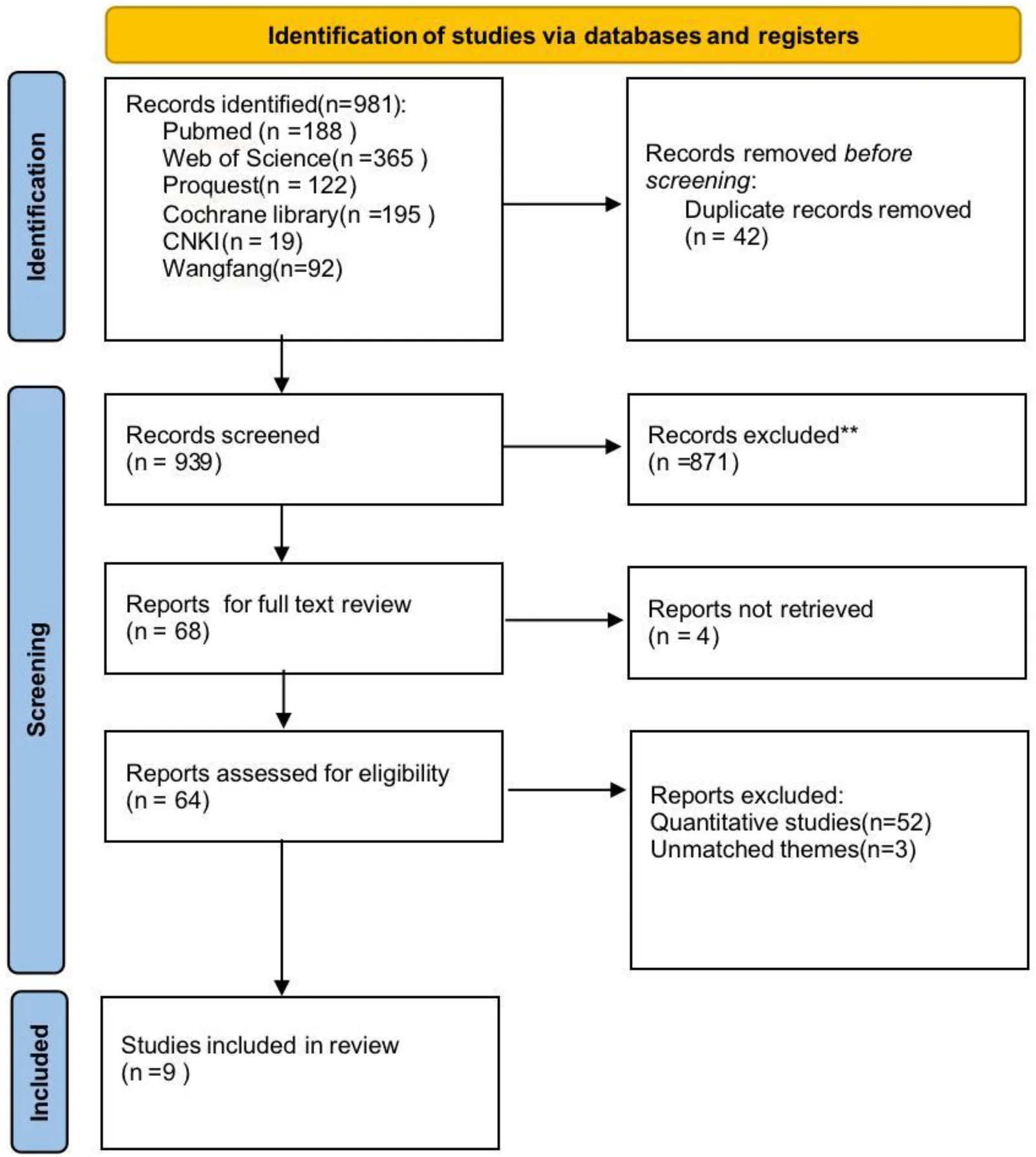
PRISMA diagram showing study selection process.

All retrieved records were screened by title and abstract after duplicate removal. Full-text articles of potentially relevant studies were then assessed for eligibility. Following the predefined inclusion criteria, nine qualitative studies were included in the final synthesis. The study selection process followed the PRISMA framework and is presented in **Figure 1**. The included studies were conducted across America (n = 5), China (n = 3), and Thailand (n = 1), and were published between 2000 and 2024. Together, they included 184 family participants, with sample sizes ranging from 8 to 31. Participants mainly consisted of mothers, fathers, siblings, and grandparents, reflecting diverse family caregiving roles across the pediatric cancer trajectory. Despite variation in study settings and qualitative designs, all included studies contributed relevant evidence on resilience, caregiving adaptation, psychosocial responses, and coping processes among family caregivers of children with cancer.

### 3.2 Methodological quality of included studies

All included studies underwent critical appraisal using the JBI Critical Appraisal Checklist for Qualitative Research. All nine studies met the pre-specified methodological quality threshold (JBI score ≥6) and were retained for synthesis. Overall, the included studies demonstrated acceptable methodological rigor and sufficient credibility to support qualitative evidence synthesis. Common strengths included clear alignment between research aims, qualitative methodology, data collection methods, and interpretation of findings. A recurrent limitation across several studies was inadequate reporting of researchers’ cultural or theoretical positioning, as well as limited discussion of the influence of the researcher–participant relationship on data generation and interpretation. However, these limitations did not substantially compromise the relevance or interpretive value of the findings (**Table 1**).

**Table 1:** Methodological quality assessment of included studies using the JBI checklist.

Study	Q1	Q2	Q3	Q4	Q5	Q6	Q7	Q8	Q9	Q10	Quality
Brody et al. (2007) (13)	Y	Y	Y	Y	Y	N	N	Y	Y	Y	Moderate
Zomerle (2015) (14)	Y	Y	Y	Y	Y	Y	Y	Y	Y	Y	High
Pierre-Louis et al.(2018) (15)	Y	Y	Y	Y	Y	N	N	Y	U	Y	Moderate
Luo et al.(2022a) (16)	Y	Y	Y	Y	Y	Y	Y	Y	Y	Y	High
Luo et al.(2022b) (17)	Y	Y	Y	Y	Y	Y	Y	Y	Y	Y	High
Suparit et al.(2023) (18)	Y	Y	Y	Y	Y	N	N	Y	Y	Y	Moderate
Huang et al.(2023) (19)	Y	Y	Y	Y	Y	Y	Y	Y	Y	Y	High
Rosenberg et al.(2023) (20)	Y	Y	Y	Y	Y	U	N	Y	Y	Y	Moderate
Gilbert et al.(2023) (21)	Y	Y	Y	Y	Y	N	N	Y	Y	Y	Moderate

Y, Yes; N, No; U, Unclear; NA, Not applicable.

### 3.3 Characteristics of included studies

The nine included studies were published between 2000 and 2024 and were conducted in America, China, and Thailand. A total of 184 participants were included, with sample sizes ranging from 8 to 31. Participants primarily consisted of parents, particularly mothers and fathers, while several studies also included siblings and grandparents. These studies reflected family caregiving experiences across different stages of the pediatric cancer trajectory, including diagnosis, active treatment, prolonged hospitalization, and survivorship. Methodologically, the included studies adopted qualitative designs such as phenomenology, descriptive qualitative research, grounded theory, and interpretive approaches. Data collection was mainly conducted through in-depth interviews or semi-structured interviews (**Table 2**).

**Table 2:** Main characteristics of the included studies.

Author (Year)	Country	Methodology	Data collection	Data analysis	Participants	Phenomenon of interest
Brody (2007) (13)	America	Qualitative	Individual interviews	Thematic analysis	8 fathers	Fathers’ experiences of family resilience during a child’s cancer treatment
Zomerlei (2015) (14)	America	Qualitative	Individual interviews	Grounded theory	16 parents	Family resilience, coping, and adaptation following pediatric cancer diagnosis
Pierre-Louis (2018) (15)	America	Qualitative	Small-group interviews	Thematic analysis	30 parents	Parenting stressors and resilience-related challenges in families of children with neurofibromatosis
Luo (2022a) (16)	China	Phenomenology	Individual interviews	Thematic analysis	23 parents	Lived experiences of resilience among parents of children with cancer
Luo (2022b) (17)	China	Qualitative	Individual interviews	Descriptive phenomenology	21 parents	Experiences of participating in a mobile-based resilience training programme
Suparit (2023) (18)	Thailand	Explanatory qualitative	Individual interviews	Thematic analysis	21 guardians	Family resilience processes among guardians caring for children with leukemia
Huang (2023) (19)	China	Qualitative	Individual interviews	Grounded theory	16 parents	Development of family resilience in families of children diagnosed with leukemia
Rosenberg (2023) (20)	America	Qualitative	Small-group interviews	Thematic analysis	18 bereaved family members	Resilience factors and adaptation among families bereaved by childhood canc
Gilbert (2023) (21)	America	Qualitative	Individual interviews	Directed content analysis	31 parents	Psychosocial risk and resilience among families during childhood cancer treatment in the COVID-19 pandemic

### 3.4 Synthesized findings

Following the JBI meta-aggregation approach, extracted findings from the included studies were grouped into conceptually similar categories and subsequently synthesized into four synthesized findings (**Table 3**). These findings collectively demonstrate that family resilience in the context of childhood cancer is not a static trait but a dynamic and evolving process shaped by emotional responses, behavioral adaptation, support systems, and coping strategies.

**Table 3:** Development of synthesized findings using the JBI meta-aggregation approach. Synthesized finding Categories Representative findings.

Synthesized finding	Categories	Representative Findings
Emotional and Cognitive Transformation in Family Resilience	Negative Emotions and Psychological Distress; Positive Emotions and Attitudinal Reconstruction	Fear, uncertainty, guilt, stigma, financial strain gradually evolved into acceptance, hope, gratitude, and cognitive reframing.
Behavioral Adaptation and Family Reorganization	Changes in Family Roles and Daily Priorities; Strengthened Family Communication and Connectedness; Meaning-Making and Life Reprioritization	Families reorganized caregiving roles, strengthened cohesion, and reconstructed life priorities.
The Role of External Support in Family Resilience	Access to Support Resources; Barriers to Support; Supporting Others and Post-Traumatic Growth; Resilience Training and Stress Management Programs	Formal and informal support promoted resilience, although access was often constrained by contextual barriers.
Coping Strategies Employed by Parents	Problem-Focused Coping; Emotion-Focused Coping	Parents adopted both practical and emotional coping strategies to manage caregiving burden.

#### 3.4.1 Synthesized finding 1: emotional and cognitive transformation in family resilience

Although fear, uncertainty, guilt, stigma, and financial strain reflected sources of adversity rather than resilience itself, these experiences constituted the contextual challenges that triggered resilience processes. Resilience emerged through families’ subsequent efforts to adapt, cognitively reframe adversity, restore hope, and reconstruct meaning.

##### 3.4.1.1 Negative emotions and psychological distress

At the beginning of the cancer trajectory, parents frequently reported shock, fear, helplessness, uncertainty, and emotional exhaustion. The diagnosis disrupted the perceived normality of family life and often generated persistent anxiety regarding treatment outcomes and survival. Financial pressure further intensified distress. One parent stated: “Still it’s a strain financially and mentally” (13). For some families, financial hardship was perceived as directly related to survival and treatment access rather than merely economic burden: “The greatest difficulty is money. Because there is no money, there is no way to treat the child. No money, no children” (19). Family reported that caregiving burden was multidimensional, involving emotional suffering, economic vulnerability, and moral distress. Some caregivers also internalized guilt and self-blame:

“At that moment, I felt like a failure. I felt quite remorseful. Because we neglected our child and didn’t bring her in for treatment sooner” (19). In addition, stigma and social misunderstanding further deepened isolation: “Some friends are afraid of this disease … My father won’t allow us to go back home either. He is worried that the neighbors in the village will discover the illness of the child … The traditional view is difficult to change…” (19).

##### 3.4.1.2 Positive emotions and attitudinal reconstruction

Despite substantial distress, many families gradually developed positive emotional adaptation. Through repeated caregiving experiences, uncertainty was increasingly replaced by acceptance, confidence, and hope.

Some caregivers reported that disease-related fear reduced over time as treatment routines became familiar: “Now I don’t treat it as a terrible disease anymore. I treat it as a cold that happens every month. Not a big deal” (16). Participants described cognitive reframing as part of their adaptation to ongoing caregiving demands. Families also expressed gratitude toward relatives and social support: “I feel very fortunate to have such good relatives and friends who help and support my family” (16). Others developed stronger internal identity and collective resilience: “It’s just a culture in our house that we’re strong, we’re faithful, we accomplish things, we conquer” (21). Humor was also used as an emotional buffer and resilience resource: “We always had a good sense of humor. We laughed a lot” (21).

#### 3.4.2 Synthesized finding 2: behavioral adaptation and family reorganization

Resilience was further reflected in practical behavioral changes that helped families reorganize priorities, maintain cohesion, and restore functional stability.

##### 3.4.2.1 Changes in family roles and daily priorities

Childhood cancer significantly altered family routines, responsibilities, and role structures. Parents often adjusted employment, caregiving duties, and household organization.

Many families intentionally reprioritized time and relational closeness. One caregiver emphasized: “We also ensure that the needs of all our children are met, not just those with cancer” (21).

##### 3.4.2.2 Strengthened family communication and connectedness

Several studies showed that adversity promoted stronger communication and mutual recognition among family members. Families increasingly valued togetherness and emotional presence: “We celebrate the little things intentionally, as we don’t know whether our daughter will be with us tomorrow” (14). Families described intentional efforts to preserve emotional meaning and connectedness despite uncertainty.

##### 3.4.2.3 Meaning-making and life reprioritization

Beyond functional adaptation, some families reconstructed life purpose through altruism and legacy-building: “To love others, to help others, and to serve others. That will be my daughter’s legacy” (20).

#### 3.4.3 Synthesized finding 3: the role of external support in family resilience

External support systems were critical for sustaining resilience. However, support was frequently perceived as fragmented, inadequate, or difficult to access.

##### 3.4.3.1 Access to support resources

Families relied on both formal and informal resources, including relatives, healthcare professionals, peer parents, schools, online communities, and religious organizations.

Support from healthcare professionals was highly valued: “The oncology team has been phenomenal … we are so grateful for the caring doctors” (14). Peer parents also contributed experiential knowledge: “Other parents told me about the side effects children might experience when using these drugs” (19).

##### 3.4.3.2 Barriers to support

Despite its importance, support access remained uneven. Some caregivers lacked family assistance: “The child’s grandparents are too old to provide more support. So now I can only be here, which means I can’t look after our other child” (19). Geographic distance also created barriers: “Our biggest challenge is finding all the support that we need when we’re not in Kansas City because when we’re in Kansas City it’s much easier” (21).

Thus, resilience was partly constrained by structural and contextual inequalities.

##### 3.4.3.3 Supporting others and post-traumatic growth

Some parents transformed suffering into altruistic motivation. One parent reflected: “All of our son’s experiences he can use to help other people somehow. He wants to be a child life specialist” (14). Family reported that resilience may extend beyond adaptation to prosocial meaning-making.

##### 3.4.3.4 Resilience training and stress management programs

Parents showed strong interest in structured resilience-based interventions, especially peer-informed and digitally accessible programs. However, caregiving burden often reduced adherence: “I received training in the hospital, but I did not have time to do it at home” (17). Future interventions must remain practical, flexible, and family-centered.

#### 3.4.4 Synthesized finding 4: coping strategies employed by parents

Families employed both problem-focused and emotion-focused coping strategies to manage uncertainty and caregiving burden.

##### 3.4.4.1 Problem-focused coping

Many parents actively sought knowledge, practical solutions, and resource optimization.

Learning from peer caregivers was common: “They (other parents) have more experience. I learn from them about how to care for the child, especially regarding diet” (16). These efforts enhanced caregiving competence and perceived control.

##### 3.4.4.2 Emotion-focused coping

Caregivers also relied on emotional regulation strategies such as acceptance, self-control, temporary withdrawal, and cognitive adjustment. However, low-resilience parents were more likely to adopt avoidant behaviors and social disengagement.

One participant reported: “Since the diagnosis, I’ve had no contact with them (previous friends); I didn’t know what to say when they reached out to me” (16). Participants described that coping quality may directly influence resilience sustainability.

Overall, coping strategies functioned as central mechanisms linking adversity exposure to adaptive family resilience.

### 3.5 Confidence in synthesized findings

Confidence in the synthesized findings was assessed using the JBI ConQual approach. Overall, all four synthesized findings achieved a moderate level of confidence. This rating reflected the generally satisfactory methodological quality of the included studies and the moderate credibility of the supporting qualitative evidence. Detailed ConQual ratings are presented in **Table 4**.

**Table 4:** ConQual summary of synthesized findings.

Synthesized finding	Dependability	Credibility	Initial ranking	Final ConQual score
Emotional and Cognitive Transformation in Family Resilience	Moderate	Moderate	High	Moderate
Behavioral Adaptation and Family Reorganization	Moderate	Moderate	High	Moderate
The Role of External Support in Family Resilience	Moderate	Moderate	High	Moderate
Coping Strategies Employed by Parents	Moderate	Moderate	High	Moderate

All synthesized findings were initially assigned a high level of confidence according to the ConQual methodology. Confidence was downgraded by one level because several included studies did not adequately report researcher reflexivity, researcher positioning, and the influence of the researcher–participant relationship. In addition, some findings were supported by a combination of unequivocal and credible evidence rather than exclusively unequivocal findings. Consequently, all synthesized findings were assigned an overall rating of moderate confidence.

## 4 Discussion

This qualitative systematic review synthesized evidence using the JBI meta-aggregation approach. Consistent with the principles of meta-aggregation, the synthesized findings were derived through aggregation of author-reported qualitative findings rather than reinterpretation of participant experiences. The review identified four interrelated dimensions of family resilience, including emotional and cognitive transformation, behavioral adaptation and family reorganization, external support, and coping strategies.

### 4.1 Resilience as a dynamic and progressive process

One major finding of this review is that family resilience developed progressively rather than emerging immediately after diagnosis. In the early stages of childhood cancer, families commonly experienced intense emotional distress, uncertainty, fear, helplessness, guilt, and financial strain. These responses were particularly evident at diagnosis and during prolonged treatment, when caregivers perceived severe disruption to normal family life. However, resilience gradually emerged through adaptation and psychological reconstruction. These findings are consistent with pediatric psycho-oncology literature, which has similarly demonstrated that caregivers experience intense psychological distress following diagnosis before gradually adapting over time (22, 23). As treatment experiences accumulated, many caregivers shifted from fear and emotional instability toward acceptance, confidence, hope, and functional adjustment. This finding is consistent with a recent qualitative systematic review, which identified hope as a fundamental psychological resource that shapes coping strategies and facilitates long-term adaptation among children with cancer and their families (24). This progression supports the understanding that resilience is not the absence of suffering, but the capacity to reorganize emotional, behavioral, and relational resources in response to adversity. This finding directly strengthens the conceptual interpretation of family resilience in pediatric oncology and addresses the need to view resilience as an evolving trajectory rather than a stable personal trait.

### 4.2 Emotional burden, financial pressure, and moral distress

This review found that caregiver burden was multidimensional. Emotional suffering was frequently intertwined with financial hardship, caregiving overload, uncertainty, and social isolation. Importantly, financial stress was not merely described as economic difficulty. Several caregivers linked financial burden to treatment continuation and child survival, indicating that economic insecurity may create profound moral distress. For families experiencing prolonged hospitalization or reduced employment capacity, financial strain intensified anxiety, helplessness, and psychological exhaustion. These findings align with previous pediatric oncology literature showing that childhood cancer affects not only the patient, but also family functioning, parental well-being, and socioeconomic stability (23). Therefore, resilience-support interventions should not focus solely on emotional coping, but also address structural caregiving burden (25).

### 4.3 Family reorganization and meaning reconstruction

Another important finding was that resilience was strongly associated with family reorganization and meaning-making. Families frequently adjusted caregiving roles, redistributed responsibilities, changed work patterns, and reprioritized emotional closeness. Rather than functioning solely around illness, some families intentionally maintained attention to siblings, preserved communication, and strengthened cohesion. Meaning reconstruction also emerged as a critical resilience mechanism. Several caregivers reported increased appreciation of life, strengthened family identity, faith, gratitude, and prosocial motivation. In some cases, adversity contributed to post-traumatic growth and new purpose. These findings suggest that resilience extends beyond coping with stress. It may involve transformation of family values, relational patterns, and existential meaning (26). Intervention studies and systematic reviews have further demonstrated that structured resilience programs can enhance family cohesion and adaptive meaning-making processes (27, 28).

### 4.4 The importance of external support systems

This review further demonstrated that resilience was closely influenced by the accessibility and quality of external support. These findings are consistent with evidence demonstrating that resilience-promoting programs and supportive care interventions significantly improve family adaptation and coping outcomes (27, 29, 30). Healthcare professionals, peer parents, relatives, schools, online communities, and spiritual support were repeatedly identified as important protective resources. Emotional reassurance, informational guidance, and practical caregiving advice helped families reduce uncertainty and improve confidence. Systematic reviews further confirm that family resilience is strongly influenced by structured psychosocial interventions and healthcare system support (28). However, support was not equally accessible. Similar findings have been reported among migrant families in pediatric oncology, where language, cultural, and healthcare access barriers further limited the utilization of supportive resources. This highlights the importance of providing equitable and culturally responsive family-centered care to enhance resilience across diverse populations (31). Geographic barriers, caregiving overload, insufficient family assistance, and fragmented psychosocial services limited support utilization. This suggests that resilience may be constrained not only by internal coping capacity, but also by broader social and healthcare inequalities (25). Therefore, family resilience should be understood as both an individual-family process and a system-supported phenomenon.

### 4.5 Coping strategies and adaptive capacity

Problem-focused and emotion-focused coping strategies were central to resilience development. Parents with stronger adaptive capacity often actively sought information, learned from healthcare teams or peer caregivers, reorganized caregiving routines, and used practical problem-solving strategies. These behaviors increased perceived control and caregiving competence. A recent latent profile analysis likewise demonstrated that higher caregiver resilience was associated with stronger family resilience, whereas limited use of social and economic resources characterized families with lower resilience. This finding underscores the importance of strengthening caregiver coping capacity while improving access to supportive resources (32). However, emotional withdrawal, avoidance, reduced social interaction, and limited help-seeking appeared more common among caregivers with lower resilience. Such strategies may temporarily reduce distress, but could weaken long-term adaptation. This finding suggests that coping style may directly influence resilience sustainability and should be considered when designing psychosocial interventions (33).

### 4.6 Integration with Walsh family resilience framework

The synthesized findings can be meaningfully interpreted through Walsh’s Family Resilience Framework, which conceptualizes resilience as a dynamic process involving family belief systems, organizational patterns, and communication and problem-solving processes. The emotional and cognitive transformation identified in this review reflects the development of adaptive belief systems through meaning-making, hope, acceptance, and positive reframing of adversity. Behavioral adaptation and family reorganization correspond to organizational processes that enable families to redistribute roles, adjust routines, and maintain functional stability during prolonged caregiving demands. The importance of external support highlights the role of social and community resources in strengthening family resilience and facilitating adaptation. Finally, the coping strategies reported by caregivers align with communication and problem-solving processes that support effective management of illness-related challenges. Collectively, these findings reinforce Walsh’s proposition that family resilience emerges through the interaction of cognitive, relational, and organizational resources rather than through individual psychological characteristics alone (34). Applying this framework provides a theoretically coherent explanation of how families adapt to childhood cancer and offers a useful foundation for developing family-centered resilience interventions in pediatric oncology settings.

### 4.7 Heterogeneity of caregiving contexts

An important consideration is the heterogeneity of caregiving contexts across the included studies. Families were represented at different stages of the childhood cancer trajectory, including diagnosis, active treatment, survivorship, and bereavement. These stages involve distinct stressors and caregiving demands, which may influence how resilience is experienced and expressed. In addition, some studies were conducted during the COVID-19 pandemic, when healthcare restrictions and social isolation may have affected family coping processes. Although common resilience processes were identified across studies, the findings should be interpreted as reflecting shared experiences across diverse contexts rather than a uniform resilience trajectory. Future research should further explore how family resilience evolves across different phases of the cancer journey and under varying clinical and social circumstances.

One included study involved caregivers of children with neurofibromatosis rather than children receiving active cancer treatment. Although neurofibromatosis differs from childhood cancer in disease trajectory and treatment intensity, it shares several resilience-relevant characteristics, including chronic uncertainty, recurrent medical surveillance, caregiver burden, and concerns regarding future malignancy risk. Nevertheless, this difference may have introduced conceptual heterogeneity and should be considered when interpreting the synthesized findings.

## 5 Clinical implications for pediatric oncology nursing

These findings have important implications for pediatric oncology nursing and family-centered care. First, routine psychosocial screening should be implemented to identify caregivers experiencing emotional distress, financial burden, or social isolation early in treatment. Second, family-centered interventions should support communication, role adaptation, and emotional coping across the entire family system rather than focusing exclusively on the child. Third, resilience-support programs should be flexible, practical, and culturally appropriate. Digital education, peer support, caregiver counseling, and structured resilience interventions may improve accessibility, especially for families experiencing long-term caregiving demands. Finally, healthcare professionals should recognize that resilience is dynamic and may fluctuate across diagnosis, treatment, survivorship, and recurrence.

## 6 Strengths and limitations

This review has several strengths. It used a systematic qualitative synthesis approach, followed PRISMA principles, and critically appraised included studies using the JBI methodology. The synthesis incorporated evidence from different countries and caregiving contexts, improving conceptual understanding of family resilience.

However, several limitations should be acknowledged. Only English and Chinese language studies were included, which may have introduced language bias. The number of included studies was relatively small, and most studies originated from limited geographic regions, particularly Asia and America. Cultural variability in resilience experiences may therefore not be fully represented. In addition, qualitative findings may reflect contextual interpretation and may not be generalizable to all caregiver populations. The included studies represented diverse caregiving contexts, including active treatment, survivorship, bereavement, and pandemic-related experiences. These contexts may influence resilience trajectories differently and should be considered when interpreting the synthesized findings. Most participants were mothers, which may have resulted in greater representation of maternal caregiving experiences compared with fathers, siblings, or extended family members.

## 7 Conclusion

This systematic review synthesized qualitative evidence on family resilience among caregivers of children with cancer and demonstrated that resilience is a dynamic, multidimensional, and evolving process shaped by emotional adaptation, family reorganization, external support, and coping strategies.

Although childhood cancer places families under considerable psychological, practical, social, and financial burden, resilience may gradually develop through cognitive reframing, strengthened family relationships, adaptive coping, and access to supportive resources. These findings suggest that resilience is not a fixed trait, but a progressive process influenced by both internal family capacity and external healthcare and social support systems.

This review highlights the importance of family-centered psychosocial support in pediatric oncology. Early emotional assessment, accessible supportive interventions, caregiver education, and resilience-oriented nursing care may help strengthen long-term adaptation and well-being among caregivers of children with cancer.

Future studies should examine family resilience longitudinally across different stages of pediatric cancer care, including diagnosis, active treatment, survivorship, relapse, and bereavement. More cross-cultural qualitative research is also needed to explore how social, economic, and cultural contexts shape resilience development. In addition, intervention studies should investigate whether structured resilience-support programs, peer-based support, and digital psychosocial strategies improve caregiver adaptation and long-term family outcomes.

## Data Availability

The original contributions presented in the study are included in the article/**Supplementary Material**. Further inquiries can be directed to the corresponding author.
