# Supplementary material for: The lived experience of resilience in families of children with cancer: a meta-aggregative synthesis of qualitative studies
Source: Front Psychiatry. 2026 Aug 21;17:1856540. doi: 10.3389/fpsyt.2026.1856540 (PMC13541751; doi:10.3389/fpsyt.2026.1856540)
Supplement: Supplementary file 1 [file Table1.docx]

**Supplementary File S1**

The electronic literature search was conducted from database inception to May 28, 2024.

No restrictions regarding publication year were applied.

Reference lists of all included studies and relevant reviews were manually screened to identify additional eligible studies.

Grey literature was searched using Google Scholar and ProQuest Dissertations & Theses Global.

**Pubmed：**

#1 ("Neoplasms"[Mesh] OR cancer*[Title/Abstract] OR neoplas*[Title/Abstract]

OR tumor*[Title/Abstract] OR tumour*[Title/Abstract]

OR leukemia*[Title/Abstract] OR leukaemia*[Title/Abstract])

#2 ("Child"[Mesh] OR "Adolescent"[Mesh]

OR child*[Title/Abstract]

OR adolescent*[Title/Abstract]

OR pediatric*[Title/Abstract]

OR paediatric*[Title/Abstract])

#3 ("Resilience, Psychological"[Mesh]

OR resilienc*[Title/Abstract]

OR "family resilience"[Title/Abstract])

#4 ("Family"[Mesh]

OR famil*[Title/Abstract]

OR parent*[Title/Abstract]

OR caregiver*[Title/Abstract]

OR guardian*[Title/Abstract])

#5 #1 AND #2 AND #3 AND #4

**Web of Science Core Collection：**

TS=(

(cancer* OR neoplas* OR tumor* OR tumour*

OR leukemia* OR leukaemia*)

AND

(child* OR adolescent* OR pediatric* OR paediatric*)

AND

(resilienc* OR "family resilience")

AND

(famil* OR parent* OR caregiver* OR guardian*)

)

**ProQuest**

AB,TI(

(cancer* OR neoplas* OR tumor* OR tumour*

OR leukemia* OR leukaemia*)

AND

(child* OR adolescent* OR pediatric* OR paediatric*)

AND

(resilienc* OR "family resilience")

AND

(famil* OR parent* OR caregiver* OR guardian*)

)

**Cochrane Library**

#1 MeSH descriptor: [Neoplasms] explode all trees

#2 cancer* OR neoplas* OR tumor*

OR tumour* OR leukemia*

OR leukaemia*:ti,ab,kw

#3 MeSH descriptor: [Child] explode all trees

#4 child* OR adolescent*

OR pediatric* OR paediatric*:ti,ab,kw

#5 resilienc* OR "family resilience":ti,ab,kw

#6 famil* OR parent*

OR caregiver* OR guardian*:ti,ab,kw

#7 (#1 OR #2)

AND

(#3 OR #4)

AND

#5

AND

#6

**China National Knowledge Infrastructure (CNKI)**

SU=

(

(儿童 OR 患儿 OR 青少年)

AND

(肿瘤 OR 癌症 OR 恶性肿瘤 OR 白血病)

AND

(韧性 OR 心理韧性 OR 抗逆力 OR 家庭韧性)

AND

(家庭 OR 父母 OR 家长 OR 照顾者 OR 监护人)

)

**Wanfang Database**

主题=

(

(儿童 OR 患儿 OR 青少年)

AND

(肿瘤 OR 癌症 OR 恶性肿瘤 OR 白血病)

AND

(韧性 OR 心理韧性 OR 抗逆力 OR 家庭韧性)

AND

(家庭 OR 父母 OR 家长 OR 照顾者 OR 监护人)

)

**Search Strategy Development：**

The search strategy was developed according to the Population–Phenomenon of Interest–Context (PICo) framework recommended for qualitative evidence synthesis. Controlled vocabulary terms (e.g., MeSH terms) and free-text keywords were combined using Boolean operators (AND/OR) and were adapted for each database.

Because resilience is frequently embedded within broader descriptions of family adaptation and caregiving experiences in qualitative pediatric oncology research, search terms were intentionally designed to maximize sensitivity by combining terms related to childhood cancer, family caregivers, and resilience. During title and abstract screening, studies describing family adaptation, coping, psychosocial adjustment, or family functioning were also carefully evaluated for potential eligibility if resilience processes were reported.

**Grey Literature Search：**

Grey literature was searched using Google Scholar and ProQuest Dissertations & Theses Global. The first 200 records sorted by relevance in Google Scholar were screened. Reference lists of all included studies and relevant reviews were manually searched to identify additional eligible studies. No additional studies meeting the eligibility criteria were identified through grey literature searching.
